# The effect of Vitamin C supplementation toward high sensitivity c-reactive protein (hsC-RP) level on male adolescent obesity in Padang

**DOI:** 10.1186/1687-9856-2013-S1-P85

**Published:** 2013-10-03

**Authors:** Nedi Hidayat, Eka Agustia Rini, Rizanda Machmud

**Affiliations:** 1 Endocrinology Division, Pediatric Health Departement of Medical Faculty Andalas University, Dr. M. Djamil Hospital Padang

**Keywords:** Adolescent, obesity, vitamin C, hsC-RP

## Background

Prevalence of obesity in children and adolescent has significantly increased, it becomes a serious problem because of the over- releasing of interleukin-6 (IL-6), disruption on the oxidative balance, risk factor of getting cardiovascular disease with typically marked by elevation of the hight sensitivity C-Reactive Protein (hsC-RP). On the way to decrease IL-6 release from the visceral adipose cell is by treating the oxidative stress with antioxidant agent, such as Vitamin C.

## Objective

To examine mean hsC-RP level on male adolescent obesity in Padang and to know the effect of Vitamin C supplementation toward hsC-RP level on male adolescent obesity in Padang.

## Method

This is an experimental double blind study with 40 samples on March until May 2011, which were divided into 2 groups. One group consumed Vitamin C 500 mg and another group consumed placebo, both twice a day for 8 weeks. The hsC-RP level is measured before and after drug consumptions. The data were analyzed with chi-square and general liniear model repeated measure, the confidence interval, p<0.05.

## Result

The mean of initial hsC-RP level is higher in the Vitamin C group than the placebo group ((2.28+1.51 vs 1.78+1.23 mg/L). At the end of the study, mean hsC-RP level were decreasing in both groups (1.09+1.13 vs 0.89+1.09 mg/L). The changing in hsC-RP level after 8 weeks isn’t significant statistically (p=0.481).

## Conclusion

The mean hsC-RP level is high and Vitamin C supplementation was not significantly decreasing hsC-RP level on male adolescent obesity in Padang.

